# Is the Loss of Previously Acquired Skills a Feature of PACS1-, PACS2- and WDR37-Related Syndromes? A Systematized Narrative Review

**DOI:** 10.3390/ijms27188207

**Published:** 2026-09-15

**Authors:** Julia del Rincón, Ana Latorre-Pellicer, Beatriz Puisac, Cristina Lucia-Campos, Marta Gil-Salvador, Laura Acero, Laura Trujillano, Pilar Pamplona, Ariadna Ayerza-Casas, Feliciano J. Ramos, Juan Pié, María Arnedo

**Affiliations:** 1 Unit of Clinical Genetics and Functional Genomics, Department of Pharmacology and Physiology, School of Medicine, University of Zaragoza, CIBERER-GCV02 and IIS-Aragon, 50009 Zaragoza, Spain; 2 Clinical and Molecular Genetics Area, Hospital Sant Joan de Déu, 08950 Barcelona, Spain; 3 Clinical and Molecular Genetics Area, Vall d’Hebron Hospital, Medicine Genetics Group, Vall d’Hebron Research Institute (VHIR), 08035 Barcelona, Spain; 4 Unit of Neurophysiology, University Hospital Lozano Blesa, 50009 Zaragoza, Spain; 5 Unit of Paediatric Cardiology, Service of Paediatrics, University Hospital Miguel Servet, 50009 Zaragoza, Spain; 6 Unit of Clinical Genetics, Service of Paediatrics, Department of Paediatrics, University Hospital Lozano Blesa, School of Medicine, University of Zaragoza, CIBERER-GCV02 and IIS-Aragon, 50009 Zaragoza, Spain

**Keywords:** PACS1, PACS2, WDR37, Schuurs-Hoeijmakers syndrome, developmental regression, neurodegeneration, loss of acquired skills, neurodevelopmental disorders, ESES, rare genetic diseases

## Abstract

PACS1, PACS2 and WDR37 form a clinical–molecular axis with overlapping neurodevelopmental phenotypes. The loss of previously acquired skills, encompassing developmental regression and neurodegenerative deterioration, has been mentioned only sporadically and has not been systematically reviewed. We conducted a systematic narrative synthesis of the literature indexed in PubMed/MEDLINE and Google Scholar, informed by relevant PRISMA principles, to identify patients in whom skill loss had been reported, assess the strength of the supporting evidence, and explore molecular mechanisms that might warrant prospective surveillance. Reports were classified as confirmed, probable, possible, or not reported according to predefined criteria. Most published reports describe patients with psychomotor delay and intellectual disability without loss of skills. Five patients with reported or suspected skill loss were identified among approximately 158 published patients, although this proportion should not be interpreted as prevalence. These cases included two patients with PACS1-related disorder, two with PACS2-related disorder, and one with WDR37-related disorder. The proposed mechanisms, including a shared autoinhibitory switch, HDAC6-dependent synaptic regulation, dynein-mediated transport and endoplasmic reticulum–mitochondria calcium handling, provide a plausible biological substrate but do not establish a causal relationship with skill loss. The available evidence does not support classifying these syndromes as primarily regressive disorders. However, the documented cases justify active surveillance and standardized longitudinal phenotypic characterization.

## 1. Introduction

Neurodevelopmental disorders (NDDs) comprise a heterogeneous group of conditions that manifest early in life and impair the acquisition of cognitive, motor, language and social skills. A substantial proportion have an identifiable genetic basis and, when considered individually, most are rare diseases. Although each disorder affects relatively few individuals, collectively they represent a relevant cause of disability worldwide [1,2].

In this context, the neurodevelopmental disorders associated with PACS1, PACS2 and WDR37 constitute a clinical–molecular axis of growing interest. At the molecular level, PACS1 and PACS2 are paralogues that arose through duplication of a common ancestral gene. They regulate intracellular protein trafficking and interact with deacetylases involved in cytoskeletal, signaling and transcriptional processes. WDR37 forms a complex with both proteins. The three genes encoding these proteins are co-expressed during early embryonic development, particularly in neural crest-derived tissues, which are important for neurodevelopment and craniofacial formation. Clinically, the associated disorders share neurodevelopmental delay/intellectual disability, a similar facial *gestalt* and, in some cases, epilepsy and systemic malformations (including coloboma and congenital heart disease) [3,4].

To date, at least 113 patients with PACS1-associated syndrome (or Schuurs-Hoeijmakers syndrome), 32 with PACS2 and 13 with WDR37 have been reported [5,6,7,8,9,10,11,12,13,14,15]. The different names used to designate these disorders are summarized in Table 1.

Isolated reports of developmental regression or progressive neurological deterioration have been described across all three syndromes [6,12,14,16,17]. Although they are scarce, they may represent under-recognized clinical manifestations or events confined to specific patient subgroups. Clarifying whether loss of previously acquired skills forms part of the natural history is relevant to affected individuals, families and clinicians because it directly informs prognostic counseling, anticipatory guidance and the interpretation of developmental change.

Although these disorders are increasingly studied, reviews examining developmental regression and neurological deterioration across the PACS1–PACS2–WDR37 axis remain scarce. To our knowledge, no previous study has assessed these features at the individual-patient level while distinguishing true loss of acquired abilities from developmental non-acquisition and systematically evaluating the strength of the available evidence. Addressing this gap, the present systematized narrative review focuses on this unresolved natural-history question and identifies the minimum longitudinal data needed for its reliable assessment.

Accordingly, we reviewed the available evidence on developmental regression and neurological deterioration across the PACS1–PACS2–WDR37 axis, characterizing reported cases, assessing the level of certainty of the available evidence, and discussing potential molecular mechanisms that may inform prospective clinical surveillance.

Although there is no universally accepted classification of developmental regression, it is generally defined as the loss of previously acquired cognitive, motor, language, social, or adaptive skills that cannot be attributed to an acute brain injury [1,18]. Developmental regression should be distinguished from neurodegeneration, in which deterioration results from the progressive and irreversible loss of neurons or brain tissue, accompanied by atrophy and pathological changes. Nevertheless, these two processes may overlap in genetic NDDs [19,20].

For the purposes of this review, four clinical scenarios of skill loss described in NDDs can be distinguished:

(i) Episodic or triggered regression, documented in genetic syndromes such as Rett syndrome, in which regression is part of the diagnostic criteria [21,22]; and in other conditions, including Phelan-McDermid or Angelman syndromes, in which skill loss may be precipitated by seizures, infections or psychiatric episodes, with variable degrees of recovery [1,18].
(ii) Regression associated with developmental and epileptic encephalopathies (DEE), in which sustained epileptiform activity disrupts neurodevelopment [23].
(iii) Progressive intellectual and neurological deterioration (PIND), a characteristic feature of many inherited metabolic disorders, including lysosomal diseases, leukodystrophies, and mitochondrial disorders, which typically follow a slowly progressive and inexorable course [24,25].
(iv) Early-onset dementia and cognitive decline, documented both in DEE and in other genetic NDDs [19,20,26,27].

The first two patterns are generally considered forms of developmental regression, as skill loss primarily reflects functional or synaptic dysfunction in a structurally preserved brain. By contrast, the latter two are more consistent with progressive structural or neurodegenerative deterioration. Nevertheless, these clinical presentations can be viewed as part of a spectrum [19,20,26].

From a pathophysiological standpoint, skill loss may result from multiple, often coexisting mechanisms [2,28,29]. Several of these mechanisms overlap with the known cellular functions of PACS1, PACS2 and WDR37, including neuronal connectivity, intracellular trafficking, and cellular energy metabolism. Consequently, these syndromes provide a particularly relevant framework for investigating the biological basis and clinical occurrence of developmental regression or neurodegeneration [3,4].

## 2. Methods

A systematized narrative literature review of skill loss in disorders of the PACS1-PACS2-WDR37 axis was performed, adapting the methodological principles of the PRISMA Statement [30], and structured according to the SPIDER framework (Table 2). PubMed/MEDLINE and Google Scholar were searched using combinations of two groups of terms linked with the Boolean operator AND, while terms within each group were combined using OR. The first block included gene/syndrome related terms (“PACS1”, “Schuurs-Hoeijmakers”, “PACS1-related disorder”, “PACS2”, “DEE66”, “PACS2-related developmental and epileptic encephalopathy”, “WDR37”, “NOCGUS”, and “neurooculocardiogenitourinary syndrome”). The second group comprised terms for loss of skills (“regression”, “developmental regression”, “loss of skills”, “skill loss”, “language loss”, “motor regression”, “cognitive decline”, “deterioration”, “ESES” and “electrical status epilepticus in sleep”). The reference lists of all included articles were also screened for additional eligible studies.

Inclusion criteria comprised all articles published in English or Spanish up to July 2026, with no lower date limit, that included a clinical description of at least one affected individual. The full set of patients described in each syndrome was retrieved (16 articles on PACS1, 11 on PACS2 and 7 on WDR37). Complementarily, molecular and mechanistic studies relevant to the PACS1–PACS2–WDR37 axis were included to provide biological context. Literature screening and data extraction were performed by a single reviewer. Given the limited and finite number of published patients and the aim of identifying all reported cases, a PRISMA flow diagram was not considered necessary.

To document developmental regression or neurodegeneration, we excluded cases where there was failure to achieve developmental milestones, stable intellectual disability, transient deterioration without documented loss of acquired ability, or deterioration more plausibly explained by other problems, including vision, hearing impairment, pain, medication, hospitalization or lack of therapeutic opportunity [1,18].

The certainty of reported skill loss was classified as follows: (i) confirmed, when longitudinal documentation demonstrated the loss of a previously acquired ability, supported by serial clinical data or concordant biomarkers; (ii) probable, when the reported loss was clinically plausible but incompletely documented; (iii) possible, when *regression* or *neurodegeneration* was mentioned without sufficient information regarding the affected domain, age at onset, temporal course, or reversibility; and (iv) not reported, when the publication contained no description of developmental regression or skill loss.

## 3. Results and Discussion

For each of the three syndromes, the literature included an initial description (two independent ones in WDR37), followed by case series, individual case reports, and narrative reviews summarizing the accumulated evidence [5,6,7,12,13,14,15,16,17,31,32,33,34,35,36,37,38,39,40,41,42,43,44,45,46,47,48,49,50,51,52].

Most reports did not describe or specifically assess the longitudinal loss of previously acquired skills. Five cases were nevertheless identified among approximately 158 published patients: two with PACS1-related disorder, two with PACS2-related disorder and one possible case with WDR37-related disorder (Table 3) [6,12,14,16,17]. This proportion should not be interpreted as an estimate of frequency because regression was not systematically assessed in most reports.

Although frequencies of some clinical features present in these syndromes have been reported descriptively in cohorts dominated by the recurrent PACS1 p.Arg203Trp and PACS2 p.Glu209Lys variants, alternative PACS1, PACS2, and WDR37 variants are represented by too few individuals to support genotype–phenotype comparisons.

### 3.1. Clinical Evidence of Skill Loss

#### 3.1.1. PACS1

PACS1-NDD was first described in 2012 based on the recurrent de novo p.Arg203Trp variant found in two patients with intellectual disability, a similar facies and variable congenital anomalies. The subsequent series published by Schuurs-Hoeijmakers et al. in 2016, comprising 19 patients, established the core clinical phenotype, characterized by intellectual disability, language impairment exceeding motor impairment, frequent but generally controllable epilepsy, hypotonia and cardiac, brain, ocular, renal and genital anomalies [5,6].

The first report of developmental regression in PACS1-NDD was described in Patient 18 of this 2016 publication by Schuurs-Hoeijmakers et al. She was a girl assessed at 11 years of age with microcephaly, short stature, complex congenital heart disease, moderate-to-severe intellectual disability (complete absence of verbal language), hypotonia, and seizures. From 10 years of age, she developed progressive gait deterioration with frequent falls, followed by an ataxic crouch gait and the need for a walker by 11 years of age. Serial brain magnetic resonance imaging (MRI) showed progressive cerebellar atrophy with childhood onset, providing structural evidence to support the observed motor skill loss [6].

A second description was published in the Italian series by Bruno et al. (2023) [16]. Patient 1 was an 8-year-old boy with bilateral clubfoot, lower-limb hypertonia and muscular hypotrophy, and psychomotor delay (he achieved assisted walking at 3 years of age and spoke his first words at 4.5 years). Following an afebrile hypertonic seizure, his parents reported a regression of motor abilities. At his most recent evaluation, at 7 years of age, he was unable to speak or walk independently. However, this report is based solely on parental observations and lacks serial clinical assessments confirming regression. Moreover, the developmental milestones being compared are not equivalent: assisted walking at 3 years and independent walking at 7 years represent different developmental abilities. Consequently, it cannot be established that a previously acquired and consolidated motor skill was truly lost [16].

No additional cases of loss of previously acquired skills were identified in the remaining literature, and no recurrent pattern of language, cognitive, motor, or adaptive skill regression has been established [5,7,31,32,33,34,35,36,37,38,39,51]. Notably, the study by Sabbagh et al. (2026), the largest and best-phenotyped PACS1 cohort published to date (24 new patients, with systematic collection of developmental milestones and epileptic profile), identified no cases of developmental regression [31]. Likewise, the only longitudinal study in PACS1-NDD (Journal et al., 2026) [51], which followed five children for 2–3.5 years, found no evidence of regression. Instead, trajectories of intelligence quotient (IQ), communication and motor function were upward, consistent with ongoing developmental progress. Brain MRI showed reduced gray and white matter volumes from early childhood but preserved trajectories, providing no evidence of a neurodegenerative process [51].

#### 3.1.2. PACS2

PACS2-DEE66 was described in an initial cohort of 14 individuals carrying the recurrent p.Glu209Lys variant. The core phenotype included neonatal or very early-onset epilepsy, global delay, variable facial dysmorphism and cerebellar dysgenesis. Unlike PACS1-NDD, neonatal-onset epilepsy is a defining clinical feature and is frequently difficult to control during the first year of life, although seizure control improves over time in some individuals [12].

The first evidence of developmental regression in PACS2 was reported in the original cohort described by Olson et al. 2018 [12]: individual 13 was a girl with a history of neonatal status epilepticus. She exhibited psychomotor delay, achieving independent walking at 36 months and using only a few isolated words at the last follow-up, together with autistic features. She developed electrical status epilepticus during sleep (ESES) at 3 years of age, accompanied by developmental regression. However, the report is limited to a single sentence, without specifying the functional domain, the temporal course, or the subsequent clinical outcome [12,52].

A second case was reported by Stoian et al. The patient was a 31-year-old man with developmental delay who achieved independent walking at approximately 2 years of age and subsequently acquired spoken language, although language development was delayed and its ultimate level of proficiency was not specified. He attended an educational center with adapted support and was able to acquire academic skills in selected subjects. He also had epilepsy, with tonic seizures beginning at 4.5 months of age, and had remained seizure-free following his last adjustment in antiseizure therapy [17].

The patient subsequently developed clinical deterioration characterized by progressive involuntary weight loss, decreased muscle strength, gait instability, strabismus, marked social withdrawal, reduced spontaneous speech and expressive language, and cognitive decline, ultimately requiring hospital admission. Neurological examination revealed nystagmus, truncal ataxia, flaccid tetraparesis, bradylalia and a reduced score of 17/30 on the Mini-Mental State Examination (MMSE). Progressive cerebral and cerebellar atrophy was found on neuroimaging. The deterioration affected multiple domains, including motor, language, cognitive, and adaptive functioning. The main limitation is the lack of detail on the previously consolidated level in some domains [17].

No additional studies have identified developmental regression as a recurrent feature of PACS2 [12,13,17,40,41,42,43,44,45,46,47].

#### 3.1.3. WDR37

WDR37-NOCGUS was described in parallel by Kanca et al. and Reis et al. as a multisystem syndrome characterized by epilepsy, colobomas or ocular dysgenesis, neurodevelopmental delay with intellectual disability, and variable congenital malformations. In many individuals the phenotype is severe from early infancy. Several nonverbal individuals with limited or absent mobility have been reported, as well as cases with infant mortality [14,15].

Proband 1 reported by Kanca et al. was a 7-year-old boy with severe intellectual disability who neither walked nor used words or signs. During the neonatal period, he presented with hypotonia and seizures, together with multisystem involvement: ocular (Peters anomaly, coloboma, glaucoma), auditory (sensorineural hearing loss), cardiac, renal and genital. The authors reported that he had experienced “significant neuroregression”; however, the affected developmental domain, the age at onset and possible triggering factors are not specified [14].

The remaining published literature on WDR37-associated disorder has not identified developmental regression as a recurrent clinical feature [48,49,50].

### 3.2. Mechanisms Potentially Related to the Pathogenesis of the Syndromes

This section summarizes the molecular mechanisms described across the PACS1-PACS2-WDR37 axis that are thought to contribute to disease pathophysiology and may help explain the biological basis of skill loss. PACS1 syndrome is almost always associated with the recurrent p.Arg203Trp (R203W) variant, whereas PACS2 is most commonly associated with the p.Glu209Lys (E209K) variant and, less frequently, with variants affecting nearby residues. WDR37 is linked to missense variants clustered predominantly in the N-terminal region. Functional studies have primarily focused on these recurrent or representative disease-associated variants and have provided important insights into the molecular mechanisms underlying each disorder. However, the molecular basis of these disorders remains incompletely understood. The findings summarized below are supported by robust experimental evidence and provide the current mechanistic framework, although they may not fully explain the observed clinical phenotypes [5,12,14,15].

#### 3.2.1. A Shared Regulatory Architecture

**Figure 1:**
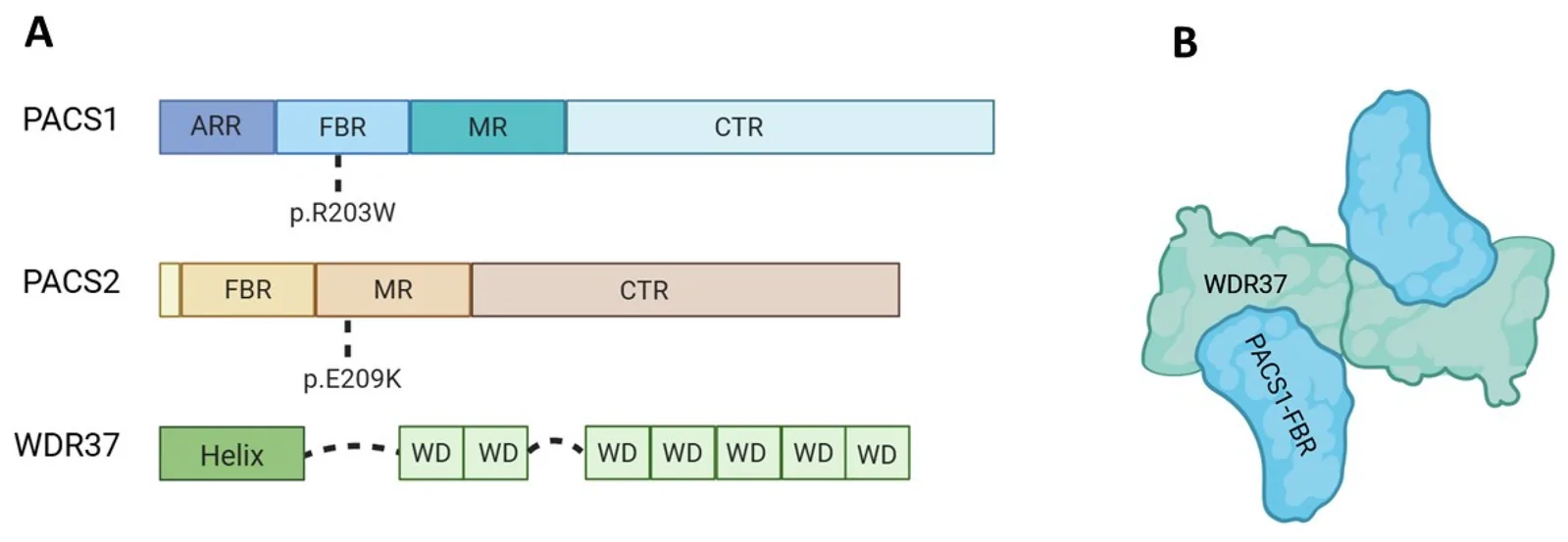
(**A**) Schematic representation of the PACS1, PACS2, and WDR37 proteins, showing their main structural domains. The pathogenic variants p.R203W in PACS1 and p.E209K in PACS2 are also indicated. ARR, atrophin-related region; FBR, furin-binding region; MR, middle region; CTR, carboxy-terminal region; WD, WD40 repeat region. (**B**) Schematic representation of the proposed interaction between PACS1 and WDR37.

PACS1 and PACS2 share a highly conserved domain architecture comprising an N-terminal region, a furin-binding region (FBR) responsible for cargo recognition, a middle region (MR), and a large C-terminal region (Figure 1A). Under physiological conditions, the MR folds back onto the FBR, maintaining the protein in an autoinhibited conformation that restricts access to client proteins. The recurrent pathogenic variants disrupt this regulatory switch from opposite regions: R203W lies within the FBR, whereas E209K lies within the MR. Both variants are considered gain-of-function alleles [12,53,54].

WDR37 forms the third component of this molecular axis by interacting with the conserved FBR of PACS1 through an interface also conserved in PACS2 (Figure 1) [55,56]. PACS1 and WDR37 are interdependent for protein stability, as disruption of either partner reduces expression of the other [55,57]. Notably, the R203W variant does not impair complex assembly, indicating that mutant PACS1 remains associated with WDR37 despite its altered autoinhibitory conformation [55].

#### 3.2.2. PACS1

Combined cellular and mouse-model studies have shown that the R203W variant aberrantly potentiates HDAC6 activity, reducing the acetylation of α-tubulin and cortactin and thereby disrupting cytoskeletal organization, Golgi integrity, and synaptic architecture. The resulting reduction in dendritic spine density and impairment of AMPA receptor-mediated synaptic transmission are consistent with a developmental synaptopathy [8]. Greater involvement of mature glutamatergic neurons and dendritic abnormalities observed in human iPSC-derived neuronal and cortical organoids further suggest vulnerability during later stages of neuronal maturation [10,58].

The R203W variant aberrantly recruits BICD2, an activating adaptor for the cytoplasmic dynein motor, and impairs dynein-dependent retrograde transport, a process required for the movement of organelles and intracellular cargo along neuronal processes. This results in Golgi and lysosomal dispersion and implicates PACS1 in defects of microtubule-dependent intracellular trafficking and long-range axonal transport [9,29]. Thus, PACS1-related neuronal dysfunction may involve both impaired synaptic organization and defective organelle transport. However, neither mechanism has been directly linked to the reported PACS1 cases with loss of skills.

#### 3.2.3. PACS2

Loss of autoinhibition caused by the E209K variant may enhance the interaction of PACS2 with client proteins involved in deacetylase-dependent signaling and calcium-channel trafficking [59,60,61,62]. However, the most distinctive function of PACS2 is its role at mitochondria-associated ER membranes (MAMs), specialized contact sites that mediate calcium transfer between the endoplasmic reticulum and mitochondria.

In cellular models, E209K reduces MAM formation and mitochondrial calcium uptake through altered CK2A1-mediated phosphorylation [63]. In experimental glutamatergic neurons, these changes alter calcium-dependent neurotransmitter release [63]. Separately, E209K increases susceptibility to stress-induced apoptosis [64]. Together, these observations suggest that some patients may be more vulnerable to neuronal dysfunction or neuronal loss under conditions of cellular stress, providing biological plausibility for neurodegenerative-type deterioration.

#### 3.2.4. WDR37

WDR37 has been identified as a critical binding partner of PACS1 [57], and previous studies have demonstrated that deletion of either PACS1 or WDR37 results in reduced expression of the other protein [55,57]. A recent study proposed that the interaction between PACS1 and WDR37 involves the FBR of PACS1 and residues 144–489 of WDR37 [55] (Figure 1B). It is proposed a WDR37 dimer structure which could interact with the FBR from PACS1. Although the structures of PACS1 and PACS2 are expected to be similar and biochemical studies have demonstrated an interaction between PACS2 and WDR37 [56], the structure of a PACS2–WDR37 complex has not yet been resolved.

The pathogenic p.Asp220Gly variant in WDR37 abolishes its interaction with PACS1 and PACS2. The loss of these interactions has been demonstrated specifically for p.Asp220Gly and should not be assumed for all pathogenic variants [56]. In addition, the PACS1-WDR37 complex regulates inositol 1,4,5-trisphosphate receptor (IP3R)-dependent calcium flux from the endoplasmic reticulum to the cytosol [57]. Disruption of these interactions may therefore compromise both complex stability and calcium homeostasis, providing a plausible molecular explanation for the phenotypic overlap observed across the PACS1–PACS2–WDR37 axis. Importantly, the individual with possible neuroregression carried p.Thr125Ile, for which no specific evidence links altered PACS binding or calcium signaling to loss of acquired skills [14,56].

### 3.3. Clinical–Mechanistic Interpretation of the Reported Loss-of-Skills Cases

PACS1, PACS2 and WDR37 disorders involve cellular pathways that could plausibly contribute to the loss of acquired skills. However, the published natural history predominantly describes neurodevelopmental disorders characterized by psychomotor delay and intellectual disability from early childhood. When reported, skill loss is uncommon, usually affects specific functional domains, and is generally poorly characterized, in contrast to syndromes in which it forms part of the core phenotype. The five documented cases identified in this review are heterogeneous and span the spectrum described in the Introduction, ranging from functional or epilepsy-associated regression to structural deterioration with a neurodegenerative-like profile.

Two cases are more consistent with functional or epileptic regression. On the one hand, we can find the ESES-associated regression reported in PACS2, the best-supported case across the axis [12]. Although the clinical description is limited, ESES is a well-recognized cause of language and cognitive regression in developmental and epileptic encephalopathies, including Landau–Kleffner syndrome [65,66]. Sustained epileptiform activity may disrupt the cortical networks underlying language, cognition and behavior, contributing to the loss of acquired skills relatively independently of the clinical seizure burden [65,67].

Although ESES-associated regression has not been reported in PACS1- or WDR37-associated disorders, epilepsy occurs in both conditions, and this mechanism should therefore be considered if developmental regression is observed. On the other hand, the motor loss triggered by a seizure described in PACS1 would also correspond to a regressive/functional episode [16].

By contrast, two cases are more compatible with neurodegenerative-like deterioration. One individual with PACS1 developed progressive gait impairment accompanied by serially documented cerebellar atrophy, representing the only case across the PACS1–PACS2–WDR37 axis with both longitudinally documented clinical decline and serial imaging evidence of structural progression. The adult patient with PACS2 showed multidomain cognitive, motor, and social decline associated with progressive cerebral and cerebellar atrophy. In both cases, the presence of structural brain changes distinguishes their clinical course from that of purely functional regression [6,17]. Progressive deterioration during adulthood has also been described in developmental and epileptic encephalopathies and other genetic neurodevelopmental disorders. For example, adults with Dravet syndrome may develop progressive cognitive and motor deterioration accompanied by parkinsonian features [26,27].

The WDR37 case cannot be confidently assigned to either category because the available clinical description is insufficient to determine the nature of the reported neuroregression [14].

At the molecular level, these clinical presentations do not converge on a single explanation. In PACS1, HDAC6-dependent synaptic and cytoskeletal dysfunction could provide a substrate for functional loss, analogous at a broad mechanistic level to developmental synaptopathies such as Rett and Phelan–McDermid syndromes. By contrast, impaired BICD2/dynein-dependent transport might be more relevant to progressive motor or cerebellar vulnerability. In PACS2, disruption of endoplasmic reticulum–mitochondria coupling, calcium homeostasis and apoptotic regulation could plausibly contribute to the adult neurodegenerative-like presentation. Unlike primary mitochondrial disorders such as Leigh syndrome or POLG-related disease, this hypothesis involves altered endoplasmic reticulum–mitochondria signaling rather than a primary respiratory-chain defect. These proposed mappings remain indirect: no molecular or functional analysis has linked any of these pathways to the clinical course of the individual patients [1,8,9,10,17,21,22,28,29,59,63,64].

Animal models illustrate the biological consequences associated with disruption of this molecular axis. PACS1 disease-model mice show dendritic and synaptic abnormalities, including dendritic varicosities, reduced spine density and impaired excitatory synaptic transmission [8]. In *C. elegans*, disease-associated PACS1/PACS2 variants alter PACS-1 localization in several cell types, including neurons [68]. WDR37 models likewise show neurological, motor and developmental abnormalities, including seizure susceptibility and impaired motor function in Drosophila and developmental abnormalities in zebrafish [14,15]. However, none of these models has demonstrated loss of an established function over time. Thus, available animal studies support neuronal vulnerability across the PACS1–PACS2–WDR37 axis but do not provide experimental evidence that developmental regression is an intrinsic feature of these disorders.

Notably, all five individuals had a history of seizures or epilepsy, although their severity, clinical course and temporal relationship with skill loss varied substantially. In the PACS2 patient with ESES, sustained epileptiform activity provides a well-established mechanism for developmental regression. In the second PACS1 case, motor decline was reported following a single afebrile seizure, raising the possibility that the event acted as a trigger, as no sustained epileptiform activity was demonstrated. The PACS1 patient with progressive cerebellar atrophy also had a history of seizures, but there is no reference to a formal epilepsy diagnosis, and their type, frequency, control and temporal relationship with deterioration were not reported. Likewise, the adult with PACS2 had longstanding epilepsy, but it was controlled at the time of later multidomain decline. The patient with WDR37 had neonatal-onset seizures, but information regarding their evolution or relationship with the reported neuroregression is insufficient. Thus, seizures may contribute to skill loss through two different pathways: sustained epileptiform activity may directly impair network function, whereas an isolated seizure could, like infection, sleep deprivation or another physiological stressor, act as a precipitating event in intrinsically vulnerable circuits [6,12,14,16,17]. However, seizures are frequent across the PACS1–PACS2–WDR37 axis. They occur in approximately 57% of individuals with PACS1-NDD, are nearly universal in published PACS2-related disorder cohorts, and affect approximately 85–95% of reported individuals with WDR37-related disorder [12,13,14,15,31,56].

Beyond their shared history of seizures, the five cases differed in gene, age at onset, affected domain, potential trigger and neuroimaging findings. We were unable to identify a clinical profile defining a subgroup at risk or a common pathogenic mechanism underlying all five cases. This heterogeneity argues against skill loss as a core feature of the PACS1–PACS2–WDR37 axis and instead supports its interpretation as an infrequent event arising through distinct, and potentially patient-specific, mechanisms.

Overall, none of these comparisons justifies considering the syndromes as primarily regressive conditions. Nevertheless, they highlight specific clinical domains in which prospective surveillance may be particularly informative.

### 3.4. Clinical Implications

From a clinical standpoint, loss of skills in individuals with PACS1, PACS2 or WDR37 disorders should neither be automatically attributed to the natural history of the syndrome, since it is not an established feature, nor dismissed solely because it appears to be uncommon.

Whenever a possible loss-of-skills event arises, the previously acquired developmental milestone, the timing of the loss and any potential triggers should be assessed in a structured manner. This evaluation should include a review of medications and recent intercurrent illness, as well as assessment of sleep, vision and hearing. In particular, the differential diagnosis of behavioral change or apparent skill loss should include unrecognized pain, a frequently overlooked cause of aggression, self-injurious behavior and irritability in individuals with intellectual disability or limited language [69].

Clinical surveillance may also be prioritized according to the gene. In PACS1 disorder, the motor and cerebellar domain appears to be the most vulnerable; therefore, deterioration in gait, balance or coordination may justify repeating brain and cerebellar MRI and comparing it with previous studies [6,16]. In PACS2 disorder, a decline in language or cognition, or any developmental plateau or regression, should prompt evaluation for clinical and subclinical seizures, including sleep EEG [12,65,67]. In contrast, no specific surveillance priorities can currently be defined for WDR37-associated disorder because the only reported case of neuroregression remains insufficiently characterized [14].

### 3.5. Implications for Research and Case Description

In order to distinguish regression from other previously described events, reports of rare diseases should incorporate a standardized description of skill loss, including the affected developmental domain, the previously acquired ability, age of acquisition, age at loss, rate of decline, potential triggers, degree of recovery, comparative neuroimaging findings, therapeutic changes, visual and auditory comorbidities and, whenever possible, adaptive or neuropsychological scales appropriate to the individual’s functional level [1].

In the absence of longitudinal studies, distinguishing developmental regression from other patterns of clinical evolution is challenging and bias may arise in both directions. Early regression may go unnoticed in individuals with severe disability or absent verbal language, whereas a static developmental course lacking precise information on milestone acquisition may be misinterpreted as progressive deterioration. An additional source of bias is the phenomenon “growth into deficit” in which the children continue to acquire new skills, but more slowly than their typically developing peers, resulting in an increasingly apparent developmental gap over time. Because standardized measures such as intelligence quotient (IQ) are interpreted relative to age-matched normative data, this widening gap may lead to declining scores that can be mistaken for true loss of previously acquired skills [1,18].

Adult cohorts are also of particular interest, since most published patients are children [5,6,7,12,13,14,15,16,17,31,32,33,34,35,36,37,38,39,40,41,42,43,44,45,46,47,48,49,50,51,52]. Improvements in medical care have increased life expectancy for individuals with neurodevelopmental disorders. It is therefore possible that more cases with degenerative or regressive symptomatology will be detected in adulthood [1].

### 3.6. Limitations

The main limitation of this review is inherent to ultra-rare diseases: the number of published patients remains small, the available cohorts are heterogeneous, some patients may have been included in more than one publication, and the depth of neurodevelopmental information varies widely between series. Moreover, most clinical reports do not provide systematic longitudinal data on the natural history of these disorders [5,6,7,12,13,14,15,16,17,31,32,33,34,35,36,37,38,39,40,41,42,43,44,45,46,47,48,49,50,51,52].

A second limitation is the scarcity of detail provided in reports describing loss of skills. Even when terms such as “regression” or “neurodegeneration” are explicitly mentioned, the affected developmental domain, timing, rate of progression, reversibility, and objective supporting investigations are rarely reported [12,14,16,17].

Third, the review may underestimate the occurrence of developmental regression because such events may remain unpublished or unrecognized, particularly in severely affected individuals, adults without a molecular diagnosis, or patients with limited verbal communication. Conversely, the absence of reported regression in a publication should be interpreted as not reported rather than absent, unless the study explicitly assessed the loss of previously acquired skills [1].

Finally, it is important to emphasize that the mechanistic evidence discussed in this review is derived primarily from cellular, animal or organoid models, which do not necessarily reproduce the dosage, tissue specificity, developmental stage or clinical context of human disease. For this reason, although the shared molecular mechanisms discussed here provide biologically plausible hypotheses, they cannot establish a causal relationship with developmental regression or neurodegenerative-like deterioration in affected individuals [8,9,10,11,12,13,14,15,48,49,50,56,59,60,61,62,63,64,70].

## 4. Conclusions

The available evidence does not support classifying PACS1-, PACS2-, or WDR37-associated disorders as primarily regressive conditions. Nevertheless, heterogeneous cases of developmental regression and skill loss have been reported across the three disorders, supporting active clinical surveillance and further longitudinal studies specifically designed to determine whether these events represent rare manifestations of the disease spectrum or an infrequent event mediated by specific complications.

In PACS1-related disorder, the documented cases of skill loss predominantly involve the motor and cerebellar domains. In PACS2-related disorder, the strongest clinical evidence is developmental regression associated with electrical status epilepticus during sleep (ESES), although an adult case with progressive cognitive and motor decline accompanied by cerebral and cerebellar atrophy also raises the possibility of a neurodegenerative-like component. In WDR37-related disorder, the single reported case of possible regression is insufficiently characterized to permit meaningful clinical interpretation.

The molecular mechanisms identified across the PACS1–PACS2–WDR37 axis overlap with pathways implicated in disorders in which developmental regression is a well-established feature, including synaptic function and neuronal network excitability, microtubule-dependent intracellular transport, and calcium signaling at endoplasmic reticulum–mitochondria contact sites. However, these mechanistic similarities should be regarded as hypotheses rather than as evidence of regression or neurodegeneration in these syndromes.

## Figures and Tables

**Table 1:** Designations used for the disorders associated with PACS1, PACS2 and WDR37.

Gene	Designations Used	Abbreviation	OMIM	Chromosomal Locus
*PACS1*	PACS1-associated neurodevelopmental disorder; Schuurs-Hoeijmakers syndrome; Schuurs-Hoeijmakers-type intellectual disability	PACS1-NDD/SHMS	#615009	11q13.1–q13.2
*PACS2*	PACS2-associated neurodevelopmental syndrome; developmental and epileptic encephalopathy type 66; early infantile epileptic encephalopathy type 66	PACS2-NDD/DEE66/EIEE66	#618067	14q32.33
*WDR37*	WDR37-associated neurodevelopmental syndrome; neurooculocardiogenitourinary syndrome; coloboma, Peters anomaly and intellectual disability syndrome	NOCGUS	#618652	10p15.3

**Table 2:** Structuring of the review question according to the SPIDER framework.

SPIDER	Component	Correspondence in This Study
S	*Sample*	Patients with disorders of the PACS1-PACS2-WDR37 axis: Schuurs-Hoeijmakers/PACS1-NDD syndrome, PACS2-related developmental and epileptic encephalopathy, and WDR37-related syndrome
PI	*Phenomenon of Interest*	Loss of previously acquired skills, in the form of developmental regression or neurodegenerative-type deterioration
D	*Design*	Case reports, clinical series and reviews that preserve individual patient information
E	*Evaluation*	Presence, domain, temporality and degree of certainty of the loss of skills
R	*Research type*	Qualitative synthesis of the evidence (systematized narrative review)

**Table 3:** Cases with evidence of regression or deterioration described in the PACS1-PACS2-WDR37 axis.

Variant	Gene/Source	Onset of Regression/Deterioration	Affected Domain(s)	Trigger	Baseline Studies (MRI/EEG)	Regression-Associated Studies (MRI/EEG)	Certainty
c.607C>T (p.Arg203Trp)	*PACS1* · Schuurs-Hoeijmakers 2016 [6], individual 18	10 years	Motor-cerebellar	No evident trigger	MRI: delayed myelination, prominent cerebellar sulci, mild cerebellar atrophy	Serial MRI: progressive cerebellar atrophy	Confirmed
c.607C>T (p.Arg203Trp)	*PACS1* · Bruno 2023 [16], patient 1	After the first seizure	Motor	Epileptic seizure	MRI: mild profile deformity and flattening of the occipital horns, mild signal alteration. EEG: background slowing, multifocal paroxysmal activity	Not reported	Probable
c.625G>A (p.Glu209Lys)	*PACS2* · Olson 2018 [12], individual 13	3 years	Global	ESES	Neonatal EEG: multifocal epileptiform activity. MRI (23 months): moderate cerebellar foliar distortion	EEG: ESES pattern	Confirmed
c.625G>A (p.Glu209Lys)	*PACS2* · Stoian 2024 [17], 31-year-old adult	Adulthood	Cognitive-motor-social	No evident trigger	Not detailed	MRI: progressive white matter disorder; severe, rapidly evolving atrophy of the brain, cerebellum and vermis; ventriculomegaly; right hippocampal atrophy. EEG: diffuse slowing + focal epileptiform activity. EMG: normal.	Confirmed
c.374C>T (p.Thr125Ile)	*WDR37* · Kanca 2019 [14], proband 1	Not specified	Not specified	Not specified	MRI (11 months): reduced brain and white matter volume, thin corpus callosum, cerebellar hypoplasia	Not reported	Possible

Abbreviations: MRI, magnetic resonance imaging; EEG, electroencephalogram; ESES, electrical status epilepticus during sleep; EMG, electromyogram. HGVS reference transcripts: PACS1, NM_018026.4; PACS2, NM_001100913.3; WDR37, NM_014023.4.

## Data Availability

No new data were created or analyzed in this study. All data discussed are available in the cited publications.

## Abbreviations

The following abbreviations are used in this manuscript:

AMPA	α-Amino-3-hydroxy-5-methyl-4-isoxazolepropionic acid
BICD2	BICD cargo adaptor 2
DEE	Developmental and epileptic encephalopathy
DEE66	Developmental and epileptic encephalopathy type 66
EEG	Electroencephalogram
EIEE66	Early infantile epileptic encephalopathy type 66
EMG	Electromyogram
ER	Endoplasmic reticulum
ESES	Electrical status epilepticus during sleep
FBR	Furin-binding region
HDAC6	Histone deacetylase 6
IP3R	Inositol 1,4,5-trisphosphate receptor
iPSC	Induced pluripotent stem cell
IQ	Intelligence quotient
MAM	Mitochondria-associated endoplasmic reticulum membranes
MMSE	Mini-Mental State Examination
MR	Middle region
MRI	Magnetic resonance imaging
NDD	Neurodevelopmental disorder
NOCGUS	Neuro-oculo-cardio-genitourinary syndrome
OMIM	Online Mendelian Inheritance in Man
PACS1	Phosphofurin acidic cluster sorting protein 1
PACS1-NDD	PACS1-associated neurodevelopmental disorder
PACS2	Phosphofurin acidic cluster sorting protein 2
PACS2-NDD	PACS2-associated neurodevelopmental disorder
PIND	Progressive intellectual and neurological deterioration
PRISMA	Preferred Reporting Items for Systematic Reviews and Meta-Analyses
SHMS	Schuurs-Hoeijmakers syndrome
SPIDER	Sample, Phenomenon of Interest, Design, Evaluation, Research type
WD40	WD40 repeat domain
WDR37	WD repeat domain 37
